# Case report: A relevant misdiagnosis: Photosensitive epilepsy mimicking a blinking tic

**DOI:** 10.3389/fped.2022.918420

**Published:** 2022-11-16

**Authors:** Francesca Burlo, Egidio Barbi, Marco Carrozzi, Caterina Zanus

**Affiliations:** ^1^ Department of Medicine, Surgery and Health Sciences, University of Trieste, Trieste, Italy; ^2^ Institute for Maternal and Child Health - IRCCS Burlo Garofolo, Trieste, Italy

**Keywords:** epilepsy, seizure, TIC, children, light

## Abstract

Blinking in children is most frequently a functional and transient symptom. Nonetheless, sometimes it is the first clinical manifestation of a neurological disorder. The differential diagnosis between voluntary actions, tics and other neurological disorders among which seizures may be challenging and misdiagnosis is common. A 6-year-old girl in good health was admitted for a recent history of bilateral eye blinking. Blinking did not interfere with the girl's activities. The patients reported that blinking seemed to be triggered by sunlight exposure and that girl sometimes seemed to be attracted by the sunlight. Ophthalmological diseases had been already excluded. The girl was addressed to our hospital for neurological consultation, as tic disease was considered the most probable hypothesis. Neurological examination was negative. In the field of differential diagnosis of photosensitive abnormal eyelid movements, the hypothesis of seizures was explored and further investigated with a video-EEG recording with light stimulation. This exam demonstrated a photoparoxysmal response (PPR) to intermittent photic stimulation with appearance on EEG of bilateral spike and polyspike waves associated with eyelid jerks. This girl suffers from generalized epilepsy with photosensitivity. Photosensitivity is a common feature of many epilepsy syndromes, mainly occurring in children and adolescents. To control the seizures, it is essential to avoid the triggering stimulus, by wearing specific glasses. Additional antiseizures treatment is often necessary, at first with valproate and levetiracetam, and ethosuximide, lamotrigine, and benzodiazepines as the second choice. Overlapping phenomenology of seizures and movement disorders is well known in paediatric clinical practice. Moreover, epilepsy and movement disorder may coexist, mainly in children. Seizures with semeiology limited to eye motor manifestations may mimic functional blinking, tics, and other motor events frequently observed in childhood. Differentiating seizures from other non-epileptic paroxysmal movements may be challenging and specialist evaluation is needed for proper treatment and prognostic counselling.

## Introduction

Unusual eyelid and ocular movements, including blinking, blepharospasms and ocular tics, are very frequent in childhood.

Blinking is a rapid movement of closing and opening of the eye. It is a normal reflex that protects the eye from dryness, bright lights, foreign objects, fingers, or other debris coming towards it. Blinking also regulates tears which nourish and cleanse the surface of the eye. It can also increase in response to pain, bright light, changes in temperature and humidity, and emotional situations. The blinking rate is low in the newborn, and it increases with growth, reaching its maximum in adolescence (1).

Blepharospasm is characterized by spasm of the orbicularis oculi muscles producing blinking or other eyelid movements like twitching (2). Similarly, ocular tics can produce simple blinking and more complex ocular movements as well, with very variable presentation.

In children, excessive blinking is most frequently a functional and even transient disease, and it resolves spontaneously (3). However, excessive blinking, photosensitivity and blepharospasms may be the first clinical manifestation of a relevant disease, such as ophthalmic or neurological disorders.

Abnormal movements of eyes and eyelids are also clinical features of different types of epileptic seizures.

As is often the case with paediatric movement disorders, overlapping phenomenology is frequent, seizures and non-epileptic paroxysmal events may be difficult to differentiate, and misdiagnosis is common. Blinking, blepharospasms, ocular tics and epileptic seizures may mimic each other, and the epileptic origin of paroxysmal ocular movements may not be properly and promptly recognized.

We report on a girl presenting light-induced tic-like eye movements and discuss the differential diagnosis that led to the diagnosis of epilepsy.

## Case report

A 6-year-old girl was admitted to the Pediatric Neurology Department to investigate a recent history of rapid bilateral upward eye movements and blinking. Blinking did not interfere with the girl's activities; she attended school regularly. Blinking was apparently triggered by the sunlight exposure, especially in summer. Moreover, parents reported that the girl seemed to be attracted by the sunlight.

The family history was remarkable for a first cousin suffering from Tourette Syndrome (TS), a sister suffering from a generalised epilepsy with photosensitivity, and a mother wearing glasses for myopia.

Ophthalmological conditions such as visual or refractory impairments, blepharitis, and conjunctival abnormalities had been already ruled out. Paediatrician suspected a diagnosis of ocular tics and addressed the family to our hospital for a neurological evaluation.

Upon admission, her physical examination was unremarkable. The eyes were neither painful nor red, and no foreign bodies were found. Neurological examination was negative. The girl showed well adapting behaviour, and normal cognitive functioning. She described as involuntary her eye movements as well as her tendency to look at the sunlight when going out. She seemed to be always aware of blinking and upward eye deviation.

**Figure 1:**
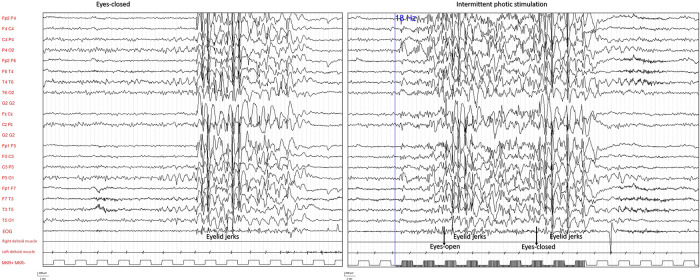
EEG polygraphic recording during wakefulness. EEG abnormalities appearing during eyes-closed resting condition (left panel) and response to intermittent photic stimulation (right panel) are shown with clinical correlate of eyelid jerks (EEG, EOG, right deltoid muscle, left deltoid muscle).

There was no evidence of other involuntary movements or neurodevelopmental abnormalities. To better define the girl's symptoms and explore photosensitivity a polygraphic video EEG recording with intermittent photic stimulation (IPS) was performed (Figure 1).

The exam demonstrated a photoparoxysmal response (PPR) as IPS activated epileptiform abnormalities consisting of bilateral high-amplitude spike and polyspike waves associated with eyelid jerks. The same electroclinical pattern was also recorded during hyperventilation and less frequently at rest. Sometimes but not exclusively and not constantly, it appeared after eye closure. These results confirmed the suspicion of a generalized epilepsy with photosensitivity. Genetic analysis with SNPs arrays was performed, without any finding of relevant mutations.

Dark, blue polarized glasses were prescribed, along with levetiracetam. Due to its ineffectiveness, the therapy was replaced with lamotrigine. Seizures’ control was still poor, requiring the add-on of valproate. Then, the girl's quality of life consistently improved, and she and her parents could face daily activities free from the fear of uncontrolled seizures.

## Discussion and conclusion

Photosensitivity and blepharospasm in school-aged children, particularly in warm seasons, are typical of vernal conjunctivitis (4), characterized by hyperaemic conjunctiva and conjunctival papillae (5). In our case, eye examination was negative, therefore this diagnosis could be excluded.

In front of a child with blinking eyes and a normal eye examination, a tic disease is usually considered first (6).

In our case, the quality of movement and its presentation during the day suggested an ocular tic. The family history of Tourette Syndrome (TS) enforced this hypothesis. Moreover, sunlight attraction was interpreted as a compulsory tic behaviour consistent with this clinical context. In fact, in the TS ocular tics are very common, and they may be the first and only clinical manifestation at the onset.

The differential diagnosis between tics, voluntary actions, and other movement disorders may be challenging, and misdiagnosis is common (7). The quality of movement must be carefully considered since it can guide clinicians towards the correct diagnosis. Tics are repetitive not rhythmic movements, they may change over time, they are often preceded by an unpleasant sensation of “urgency to do”. They occur intermittently and can, to some degree, be suppressed by effort of will (8). This sets them apart from the gaze impersistence of chorea, the incessant, multi-directional eye “dancing” of opsoclonus, dystonic oculogyric crises and from tonic or clonic eye or eyelid movements of seizures (9).

Concerning epilepsy, blinking may be part of very brief seizures apparently only involving the eyes with rapid tic-like movements that can be misleading for diagnosis. Particularly, blinking may be observed in eyelid myoclonia which is an ictal phenomenon characterized by rapid, jerky blinking immediately after eye closure associated with mild upward gaze deviation (8). Eyelid myoclonia can be differentiated from tics by the rapid frequency of blinking, transient unresponsiveness, and photosensitivity. However, during eyelid myoclonia, awareness may be intact or mildly impaired, and awareness impairment may be subtle and not always recognizable.

In the last proposal of epilepsy syndromes classification by the International League Against Epilepsy (ILAE) (10), eyelid myoclonia is described as consisting of brief, repetitive and often rhythmic, 3–6 Hz myoclonic jerks of the eyelids, with simultaneous upward deviation of the eyeballs. These seizures are very brief (typically 1–3 s, always <6 s) and occur multiple times each day, often multiple times per hour. They are typically induced by involuntary or voluntary slow eye closure or exposure to bright light or sunlight (10).

Moreover, paroxysmal eyelid movements (PEM) have been reported as not-epileptic movements in patients with generalized epilepsies and photosensitivity, characterized by eyelid closure, eyes upturning and rapid eyelid flutter, with retained awareness. These episodes do not present any EEG abnormalities and are not triggered by hyperventilation or photic stimulation (11). A coexistence of PEM, photoparoxysmal EEG responses, increased blinking and epileptic eyelid myoclonia have been already reported, suggesting an underlying dysfunction involving cortical-subcortical neural networks and a possible genetic relationship between PEM and epilepsy (12).

Photosensitive epilepsy is a broad term comprising all forms of heterogeneous epilepsies in which seizures are triggered by photic stimulation, that is the exposure to either flashing lights or certain visual patterns. Photosensitivity occurs in about 2%–5% of all forms of epilepsy. In the case of a seizure consistently elicited by a specific stimulus, the term of reflex seizure can be used. Light-induced seizures are the most frequently recorded, accounting for 5% of all 6.5% of reflex seizures (13, 14). Even though light-induced epileptic seizures have been documented since the mid-nineteenth century, there are still problems with classifying these types of seizures (15). The literature on these seizures is limited and the multitude of terms used makes it difficult to classify them unequivocally. As specified in the last ILAE classification, for the definition of reflex seizure the triggering stimulus must have a high likelihood of eliciting a seizure, in contrast to a stimulus that may facilitate epileptiform abnormality (such as photoparoxysmal responses on EEG) or evoke a seizure, but not consistently (10).

Photosensitivity is reported as a common feature of many epilepsy syndromes, and the Task Force concluded that disorders associated with photosensitivity were too diverse, when grouped, to satisfy criteria for an epilepsy syndrome (10).

Photosensitive seizures are widespread in children and adolescents, especially those with generalized epilepsy and with certain epilepsy syndromes, such as juvenile myoclonic epilepsy and epilepsy with eyelid myoclonia.

During childhood and adolescence, the period when epilepsy most frequently begins (about 75% of cases), the PPR to intermittent light stimulation is most pronounced in EEG (16–18). Therefore, children and adolescents constitute the most numerous group at-risk for the occurrence of reflex seizures or epilepsy syndromes with PPR.

Sometimes, patients can induce their seizures with light stimulation (19).

Self-induced seizures produced by stimulation of natural light are rare and self-induction is a mode of seizure precipitation employed by either intellectually disabled or healthy photosensitive individuals (20).

In the patients with prominent photic induction, eyelid myoclonia (with or without absence), absence or myoclonic seizures may be associated with behaviours such as facing a light source and hand-waving in front of the eyes, rubbing the forehead, going up close to television, or using other means to create a flickering effect of light (21, 22).

The diagnosis of self-induction in photosensitive patients with normal intelligence requires careful history-taking. Long-term video-EEG monitoring is sometimes required because self-induction may occur after the technician leaves the room or after the EEG is discontinued (23, 24).

There has been considerable speculation on the motivation for self-induction. Theories include wilful avoidance of unpleasant situations, seeking pleasure, relief of tension, addiction and compulsion (25).

Children and adolescents in particular may use their reflex phenomenon to evoke pleasant, relaxing feelings as a result of the epileptiform discharges (20).

A particular sun-seeking behavior is described in the Sunflower Syndrome (SFS), a photosensitive epilepsy now described as a rare childhood-onset generalized epilepsy characterized by photosensitivity, heliotropism, and drug resistant highly stereotyped seizure (25). Patients with SFS are commonly females, are extremely photosensitive and only rarely suffer from other form of seizure (25). Typically, the patient turns his face towards the sun, waves his hand in front of the face, rapidly blinks, or performs other movements that create a similar flicker effect. Patients can show some attraction to the sun, which may be misdiagnosed as a behavioural problem, particularly if other symptoms of a neurodevelopmental disorder are present. Some diagnostic issues of SFS are currently debated, and to date it is not conclusively defined if the handwaving motion is part of the seizure or a mechanism of self-induction (26).

For photosensitive seizures the avoidance of the triggering stimulus plays an essential therapeutic role. The use of dark, polarized, or specially tinted blue glasses seems to be also effective. However, additional antiepileptic prophylaxis is often necessary. To treat visually induced seizures, the first-line treatment is valproate and levetiracetam, while ethosuximide, lamotrigine, and benzodiazepines are the second choice.

Seizures of the SFS are often refractory to traditional anticonvulsant medications, and patients benefit mainly from non-pharmacological behavioral interventions, such as an intensive use of sunglasses or focusing on other activities to reduce the handwaving episodes (26–29). Data on the long-term prognosis of SFS are scant and clear evidence over best treatment strategies is lacking (29).

In their cases series of patients with SFS, Belcastro et al. described a high rate of drug-resistance and underline that lenses seem to have a powerful potential role for the management of this condition (25).

In our case, video-EEG recording with IPS was crucial for diagnosis, with evidence of epileptic seizures favoured by light stimulation consistent with diagnosis of generalized epilepsy with photosensitivity. Epileptic ocular movements were not constantly induced by eye closure, and they also occurred at rest and during hyperventilation. The light stimulation was the most relevant triggering stimulus. Absence seizures were never recorded, therefore diagnostic criteria for Jeavons syndrome nor eyelid myoclonia were not completely met. The patient showed a kind of sun-attracted behaviour, as frequently described in these forms of epilepsy. Nonetheless, SFS's typical complex motor behavior was never observed.

Seizures were initially refractory to the first line treatment, while they were successfully controlled by the use of either lens or a two-drugs therapy. In particular, the use of blue lenses was consistently effective in the seizure's control (25).

In conclusion, overlapping phenomenology of seizures and movement disorders is well known in paediatric clinical practice. Moreover, epilepsy and movement disorder may coexist, mainly in children. Seizures with semeiology limited to eye motor manifestations may mimic functional blinking, tics, and other motor events frequently observed in childhood. Paediatricians should be aware that blinking may be part of a more complex eye movement disorder that can be epileptic in origin. Of course, simpler, and more frequent diagnosis, such as ophthalmological conditions, must be investigated first. Nonetheless, epilepsy must be suspected and ruled out, particularly in the case of photosensitive movements. Differentiating seizures from other non-epileptic paroxysmal movements may be challenging and specialist evaluation is needed for proper treatment and prognostic counselling.

## Data Availability

The original contributions presented in the study are included in the article/Supplementary Material, further inquiries can be directed to the corresponding author/s.
